# Bile cholesterol and viscosity, the keys to discriminating adenomatous polyps from cholesterol polyps by a novel predictive scoring model

**DOI:** 10.1186/s12876-020-01414-9

**Published:** 2020-08-14

**Authors:** Eun-young Kim, Tae-ho Hong

**Affiliations:** 1 Division of Trauma and Surgical Critical Care, Department of Surgery, Seoul St. Mary’s Hospital, College of Medicine, The Catholic University of Korea, Seoul, Republic of Korea; 2 Division of Hepato-biliary and Pancreas Surgery, Department of Surgery, Seoul St. Mary’s Hospital, College of Medicine, The Catholic University of Korea, 222, Banpo-daero, Seocho-gu, Seoul, 06591 Republic of Korea

**Keywords:** Adenoma polyp, Bile, Cholesterol polyp, Gallbladder polyp, Predicting model

## Abstract

**Background:**

Adenomatous gallbladder polyps, premalignant lesions of the gallbladder, have fatal outcomes, whereas cholesterol polyps have benign features. Herein, we proposed a novel, predictive scoring model of adenomatous polyps to distinguish them from cholesterol polyps, by analyzing bile components and bile viscosity.

**Methods:**

Patients with gallbladder polyp pathologically confirmed after cholecystectomies were analyzed. After dividing patients into two groups (adenomatous or cholesterol polyps), the clinicopathologic profiles and bile nature, including components and viscosity were compared and a predictive scoring model for adenomatous polyps was assessed.

**Results:**

Eleven adenomatous polyps and 96 cholesterol polyps were analyzed. The variables significantly associated with adenomatous polyps were age > 55 years (*OR* = 23.550, *p = 0.020*), bile viscosity< 7.5 s^− 1^ (*OR* = 22.539, *p = 0.012*), and bile cholesterol< 414.5 mg/dl (*OR* = 10.004, *p = 0.023*) and the points for each variable in the predictive scoring model were allocated as 3, 3, and 2, respectively. Final scores ranged from 0 to 8 points and the best performance of model at a cutoff of ≥6 points had 90.9% of sensitivity and 80.2% of specificity.

**Conclusions:**

Bile viscosity and bile cholesterol accompanied by age were revealed as significant predictors of adenomatous polyps, distinguishing them from cholesterol polyps of gallbladder. It can be the cornerstone for creating accurate guidelines for preoperatively determining treatment strategies of gallbladder polyps.

## Background

Polypoid lesions of the gallbladder (GBP) are defined as any raised lesion of the mucosal surface of the gallbladder. Recently, with the advancement in sonographic tools and increased health screening examinations, the incidence of GBP has increased gradually from 5 to 10% in adults [1] and GBPs are found in 0.6–4% of the cholecystectomy specimens worldwide [2].

The majority of GBPs are asymptomatic benign polyps which are classified as pseudopolyps, and most of them are cholesterol polyps. However, about 2% of the GBPs have malignant potential and most of which are adenomas. Unfortunately, these premalignant lesions could proceed to gallbladder cancer, usually with a dismal prognosis as the stage advances. Only about 15–47% of the patients have a chance of curative resection and a low 5-year survival rate of less than 5–12% [3, 4]. Thus, it is crucial to early and accurately distinguish between adenomas that require true surgical resection and cholesterol polyps, which account for a significant proportion of the GBPs. According to previously published guidelines from various committees [5], polypoid lesions with a size of 10 mm or more on ultrasound examination are considered to have malignant potential and cholecystectomy is recommended. However, for polyps larger than 10 mm in size, the morphological size-based classification has limitations in accurately distinguishing adenomatous polyps from cholesterol polyps that do not require cholecystectomy. However, cholesterol polyps are generally characterized by cholesterol-laden foamy histiocytes, a lipid component that is very distinct from the components of adenomatous polyps. Previous studies reported that cholesterol polyps were significantly associated with alterations in bile acid secretion that is different from the mechanism of adenoma formation [6]. Therefore, the differences in these polyp traits and bile components can be helpful to more specifically distinguish premalignant lesions from benign lesions.

Herein, we explored the characteristics of cholesterol polyps and adenomas based on biochemical parameters that included the bile nature and derived a predictive model of premalignant polyps to establish appropriate strategies for the management of GBP.

## Methods

### Study population and medical data collection

This study was approved and carefully monitored by our Institutional Review Board. From March 2017 to March 2018, all patients admitted to our institution to undergo cholecystectomies due to any gallbladder lesions underwent bile acid sampling during specimen retrieval after the cholecystectomy and component analysis of the bile acid was performed. Among the patients diagnosed with polypoid lesions of the gallbladder larger than 1 cm, patients with cholesterol polyps or adenomas identified on the permanent pathology test were enrolled for analysis and retrospectively reviewed. After cholecystectomy, histopathological examinations were carried out by a skilled histopathologist in our institution and the preoperative ultrasound images were also assessed by a qualified radiologist. The GBPs were classified as benign or premalignant polypoid lesions according to the histopathological features. The benign polyps were mostly cholesterol polyps, and some non-cholesterol polyps consisted of adenomyomatosis, hyperplastic polyps and inflammatory polyps. Premalignant lesions are defined as dysplasia of the gallbladder polyp cells, which in most cases, are adenomas of the gallbladder. If multiple pathologic diagnoses co-existed in one specimen, the overall subtype of the polyp was determined according to the most dysplastic subtype histologically. The size of the polyp was measured from the gross pathology. If there were a variety of sizes or multiple polyps, the largest size dimension was recorded. Cholecystectomies which were performed as part of primary non-gallbladder surgery, such as hepatectomies or pancreatectomies, were excluded from the study. Rarely, cases diagnosed as non-cholesterol benign lesions were also excluded from the analysis.

All demographic information and medical data were prospectively collected from patients and compared between the groups of patients with cholesterol polyps and adenomatous polyps. Blood chemistry data were routinely obtained twice, once at admission and the day after surgery. It included liver profile tests, such as alanine aminotransferase, aspartate aminotransferase, and total bilirubin, and lipid profiles, including triglycerides (TG), total cholesterol, high-density lipoprotein, and low-density lipoprotein. Tumor markers, such as carcinoembryonic antigen and carbohydrate antigen 19–9, were also obtained from the patients at the time of admission.

### Bile collection and analysis of bile acid composition

We analyzed the bile samples in the two types of polyps to compare the characteristics between cholesterol polyps and adenomas from the study patients, all of which underwent cholecystectomies and bile sampling. While retrieving the specimen from the abdominal cavity, about 10 ml of bile acid was extracted from the resected gallbladder of each patient directly by needle aspiration. To prepare the bile, after centrifugation at 15,000 rpm for 5 min at 4 °C, 100 μL of supernatant bile fluid was added to 900 μL of 0.01% formic acid solution and 10 μg/mL of internal standard, then gently vortexed for 30 s. The bile sample was stored at − 30 °C prior to analysis and the cholesterol content was determined colorimetrically by the Liebermann-Burchard reaction after a double extraction of a 1 ml methanolic bile sample with petroleum ether as described in a study by *Abell* et al. [7]. The bile salts were analyzed by the modified 3-α-hydroxysteroid dehydrogenase method as described by *Talalay* et al. [8]. The total protein content of the bile was assessed by the Lowry assay after the purification of biliary proteins as described by *Jüngst D* et al. [9].

### Determination of the viscosity of bile acid

Three milliliters of bile sample was required for all viscosity assays and the samples were centrifuged at 3800 rpm for 5 min. The bile supernatant was separated from sedimentation products, such as sludge or microconcrements, and preincubated in a water bath at 37 °C for 15 min. Bile viscosity was measured only in the upper layer of bile and was not influenced by sediments, such as suspended solids or sludge. Bile viscosity was measured by an automated scanning capillary tube viscosity measuring instrument, Hemovister® (Hemovister, Ubiosis, Seongnam, Korea). A rotation viscosimeter allows for accurate measurements of viscosity in both Newtonian and non-Newtonian fluids. To obtain a rapid, standardized measurement within applied high shear rates (from 1 to 1000 s^− 1^), a computer program was used to enable measurements in a single sample within 5 min. These measurements were repeated twice after intervals of 60 s at 37 °C.

### Statistical analysis

All statistical analyses were conducted using SPSS statistical package software version 21.0 for Windows (SPSS, Inc., Chicago, IL, USA). The categorical variables were calculated using Fisher’s exact test or Chi-squared tests. The continuous data are presented as mean ± standard deviation. The overall differences were analyzed with Student’s *t*-tests. The descriptive statistics are presented as mean ± standard deviation. Whether the variables were normally distributed was tested using the Kolmogorov-Smirnov test, and in the case of variables that were not normally distributed, a nonparametric test was performed using the Mann-Whitney test. Differences were regarded as statistically significant at *p* < 0.05. The primary endpoint of the current study was the identification of risk factors for premalignant GBP patients with adenoma polyps compared to patients with benign cholesterol polyps. In the multiple regression analysis, only significant variables in the univariate analysis were assessed using Cox’s proportional hazard model. The receiver-operating characteristic (ROC) curve analysis assessed the cutoff value of significant risk factors associated with premalignant polypoid lesions of the gallbladder to suggest precise guidelines for the management of polypoid gallbladder lesions preoperatively. Hosmer and Lemeshow tests were conducted to measure the goodness-of-fit of the data, with *p*-values of > 0.05 indicating an acceptable calibration. The accuracy of each model was analyzed using c-statistic as a measure of discrimination.

## Results

### Patient enrollment and the prevalence of GBP in the study population

**Fig. 1:**
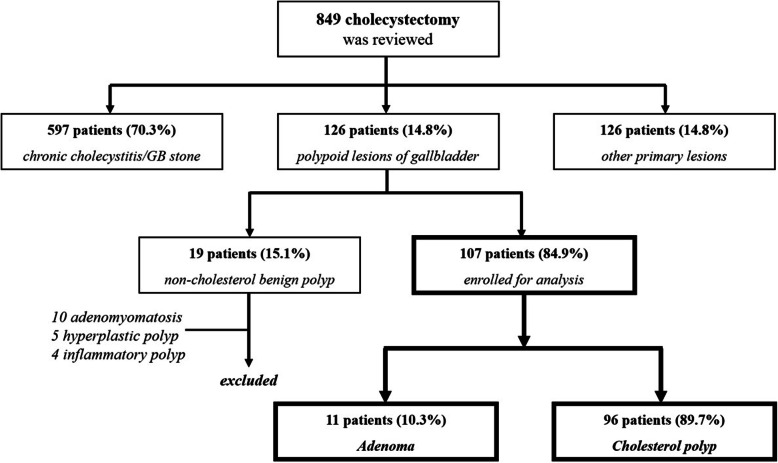
Schematic diagram of patient enrollment. A total of 107 patients with gallbladder polypoid lesions were finally analyzed and among them, 11 patients had adenomas and 96 patients had cholesterol polyps

During the study period, a total of 849 patients underwent cholecystectomies at our institution. On the permanent pathology, 597 patients (70.3%) had chronic cholecystitis or cholelithiasis, 126 patients (14.8%) had other primary lesions (not gallbladder), and 126 patients (14.8%) had any type of GBP. Figure 1 presents the outline of patient selection and final diagnoses on the pathologic reports. After excluding 19 patients with non-cholesterol benign polyps (10 with adenomyomatosis, five with hyperplastic polyps, and four with inflammatory polyps), a total of 107 patients, 96 patients with cholesterol polyps (89.7%) and 11 patients with adenomas (10.3%), were enrolled for analysis. Of the enrolled patients, the mean age was 50 years (range, 20–82 years) and there were 40 (37.4%) male patients. The overall prevalence of cholesterol polyps among the GBP patients was 76.2% (96/126) and the prevalence of adenomas was 8.7% (11/126).

### Analysis of the risk factors of premalignant GBP: adenomatous versus cholesterol polyps

**Fig. 2:**
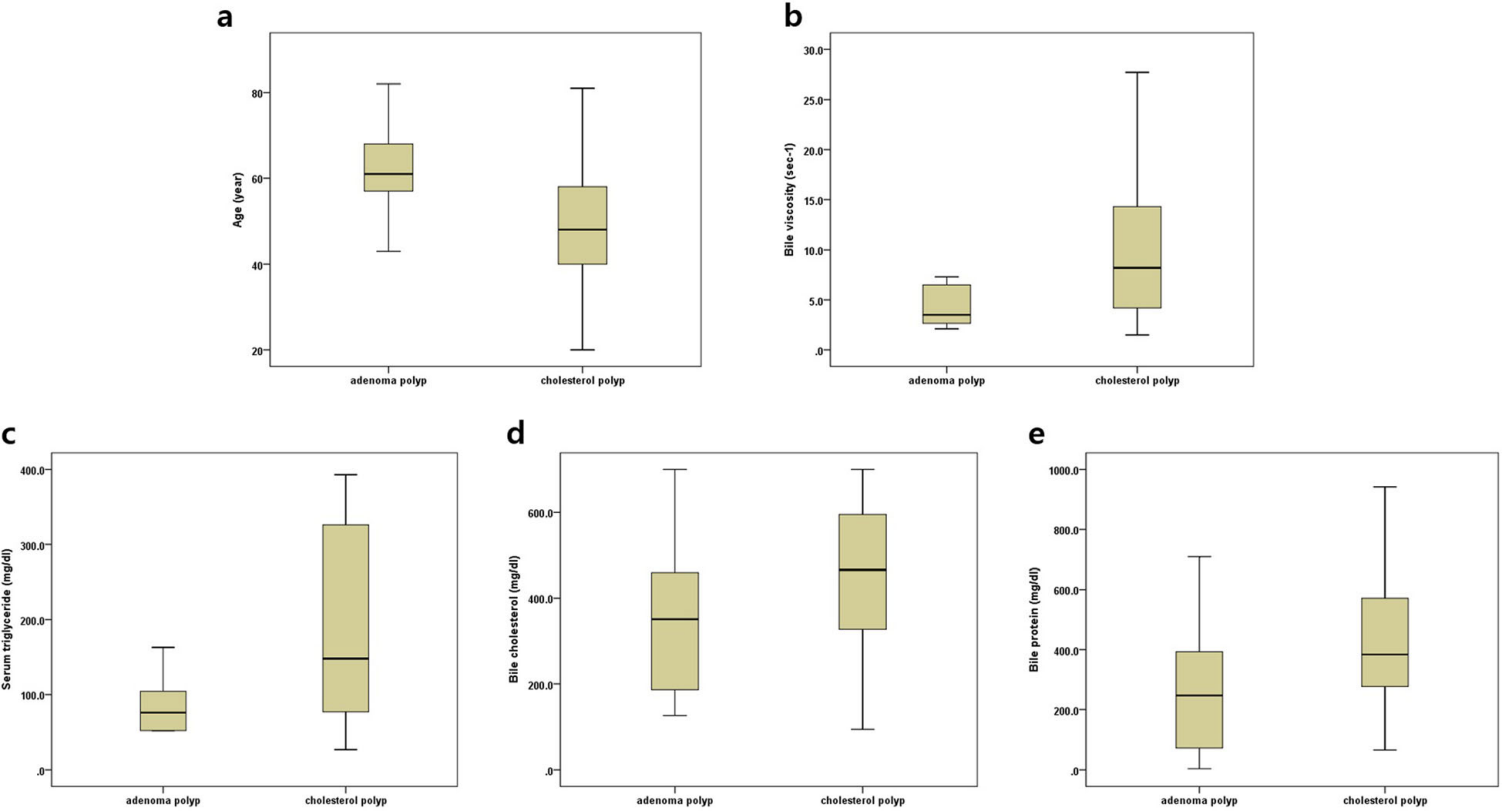
Distribution given in the square plot of the detected lesions by parameters that were significant in univariate analysis. **a** Age, **b** bile viscosity, **c** serum triglyceride, **d** bile cholesterol and, **e** bile protein

According to the final histopathology of GBP, the baseline demographics and the serum and bile acid analysis between the adenoma and cholesterol group were compared and the results are summarized in Table 1 and Fig. 2. The mean patient age was significantly older in the adenoma group than in the cholesterol group (61.7 years in the adenoma patients versus 48.7 years in the cholesterol polyp patients, *p = 0.002*). There were significant differences only in the plasma TG levels (86.3 ± 37.9 mg/dl in the adenomas versus 193.3 ± 129.9 mg/dl in the cholesterol polyps, *p < 0.001)*. In the analysis of polyp bile acid components, the levels of bile cholesterol and bile protein were significantly lower in the adenoma group than the cholesterol group (358.5 ± 209.6 mg/dl and 276.8 ± 229.7 mg/dl in the adenoma group versus 466.8 ± 160.1 mg/dl and 494.1 ± 339.3 mg/dl in the cholesterol group, *p = 0.042* and *p = 0.041*, respectively), whereas there were similar results in the levels of bile acid and bilirubin. In the hydrodynamic aspect of the bile acids, the bile acid viscosity was higher in the cholesterol polyps than in the adenomas with statistical significance. (5.3 ± 4.5 s-^1^ in the adenomas versus 10.2 ± 8.1 s-^1^ in the cholesterol polyps, *p = 0.006*).

**Table 1:** Baseline demographic characteristics and laboratory parameters between the adenoma group and cholesterol polyp group

	Adenoma (***n*** = 11)	Cholesterol polyp (***n*** = 96)	***p*** value
Age, years	61.7 (43–82)	48.7 (20–81)	*0.002*
Sex (Male/Female), *n*	2 / 9	38 / 58	*0.204*
Body mass index	24.2 ± 3.9	24.3 ± 3.8	*0.971*
Blood chemistry parameters			
*serum* AST	20.8 ± 4.3	26.7 ± 20	*0.331*
*serum* ALT	17.6 ± 7.7	24.3 ± 15.6	*0.169*
*serum* bilirubin	0.74 ± 0.4	0.73 ± 0.35	*0.147*
*serum* HDL	57.3 ± 17.9	51 ± 13.2	*0.221*
*serum* LDL	113.9 ± 31.6	107.8 ± 29.4	*0.584*
*serum* total cholesterol	196.9 ± 33.4	186.7 ± 36.6	*0.455*
*serum* TG	86.3 ± 37.9	193.3 ± 129.9	*< 0.001*
Bile acid analysis			
Bile viscosity	5.3 ± 4.5	10.2 ± 8.1	*0.006*
Bile acid^a^	12,366.8 ± 5858.8	13,945 ± 3720.5	*0.326*
*bile* bilirubin	120.8 ± 77.5	196.8 ± 148.1	*0.098*
*bile* total cholesterol	358.5 ± 209.6	466.8 ± 160.1	*0.042*
*bile* protein	276.8 ± 229.7	494.1 ± 339.3	*0.041*

Results are expressed in mean ± standard deviation

*CEA* aspartate aminotransferase, *CA19–9* alanine aminotransferase, *AST* aspartate aminotransferase, *ALT* alanine aminotransferase, *HDL* high-density lipoprotein, *LDL* low-density lipoprotein, *TG* triglyceride

^a^In the case of bile acids, the sample did not have a normal distribution, thus a non-parametric test was performed using the Mann-Whitney test

Before performing multivariate analysis to determine the risk factors for premalignant GBP, the cutoff values of each significant parameter in univariate analysis were set and included 55 years of age, 7.5 s-^1^ bile viscosity, 414.5 mg/dl bile cholesterol, 210.5 mg/dl bile protein, and 140 mg/dl serum TG. In multivariate logistic analysis, age older than 55 years, bile viscosity less than 7.5 s-^1^, and bile cholesterol less than 414.5 mg/dl were found to be independent predictive variables for adenomatous polyps (odds ratio (OR) = 23.550, 95% confidence interval (CI): 1.658–334.587, *p* = 0.020; *OR* = 22.539, 95% CI: 1.962–258.906, *p* = 0.012; and *OR* = 10.004, 95% CI: 1.375–72.816, *p* = 0.023, respectively), as presented in Table 2. The logistic regression model including these significant factors had a c-static of 1.109 according to the Hosmer-Lemeshow test, indicating acceptable calibration with high accuracy of 92.5%.

**Table 2:** Multivariate logistic regression analysis of risk factors associated with adenoma gallbladder polyp

Variable	β	Odds ratio	95% CI	***p-value***
Age > 55	3.159	23.550	1.658–334.587	*0.020*
Bile viscosity < 7.5 s-^1^	3.115	22.539	1.962–258.906	*0.012*
Bile cholesterol < 414.5 mg/dl	2.303	10.004	1.375–72.816	*0.023*

### Relevant predictive models for premalignant GBPs

**Fig. 3:**
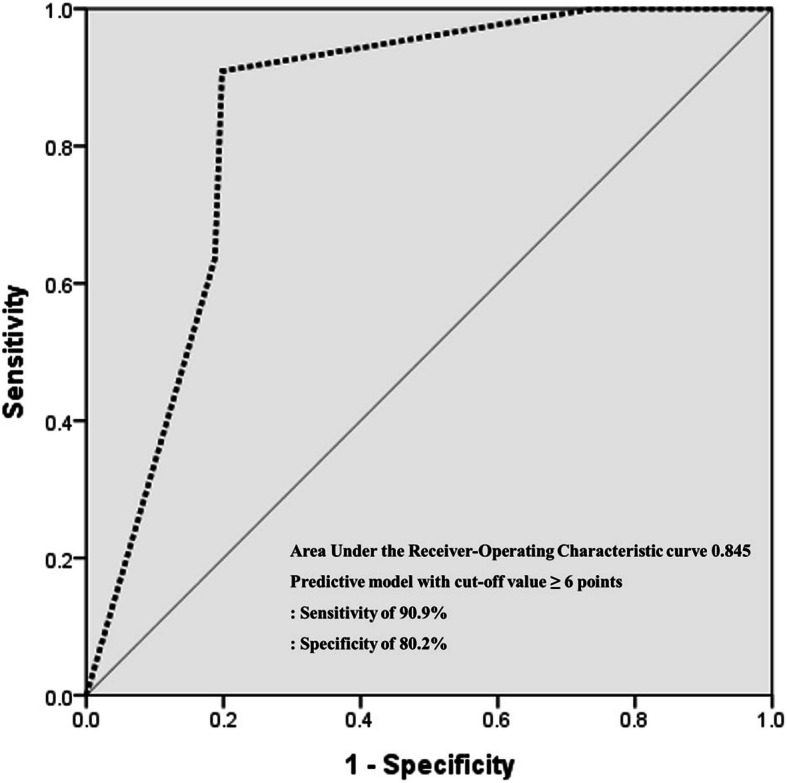
Predictive scoring model of adenomatous polyps of the gallbladder. This model showed an AUROC (area under the receiver-operating characteristic curve) of 0.845 with 90.9% sensitivity and 80.2% specificity at a cutoff of ≥6 points. The point assigned to the parameters were: age > 55 years, 3 points; bile viscosity < 7.5 s^− 1^, 3 points; and bile cholesterol < 414.5 mg/dl, 2 points

**Fig. 4:**
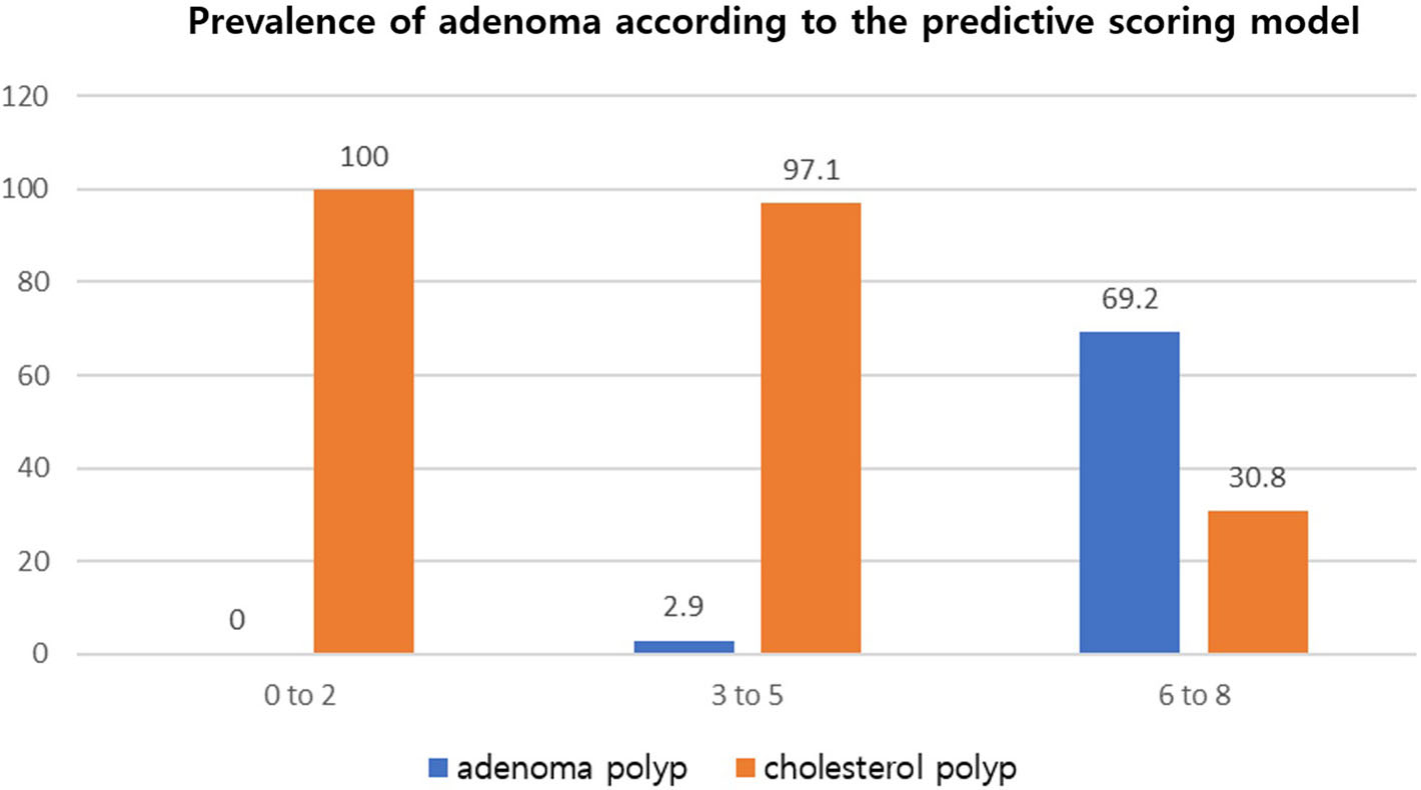
Correlation between points in the predictive model and the possibility of adenomatous polyps of the gallbladder. The probability of adenomatous polyps of the gallbladder increased progressively with scores from 0 to 2, to 3 to 5, and to 6 to 8 points with the following percentages: 0% (*n* = 0/26), 2.9% (*n* = 2/ 68), and 69.2% (*n* = 9/13), respectively (Goodman & Kruskall Gamma *p < 0.001*)

Using the significant variables from multivariate logistic regression analysis, we developed a predictive scoring model for adenomatous polyps that would distinguish them from cholesterol polyps. Points in the predictive scoring model for adenomas were allocated as shown in Table 3: age > 55 years (yes = 3 points, no = 0 points), bile viscosity < 7.5 s-^1^ (yes = 3 points, no = 0 points), and bile cholesterol < 414.5 mg/dl (yes = 2 points, no = 0 points). Finally, the points from this predictive model ranged from 0 to 8 and the best performance of the model at a cutoff of ≥6 points had a sensitivity of 90.9% and a specificity of 80.2%, with an area under the ROC curve of 0.845 (Fig. 3). The probability of adenoma polyps of the gallbladder increased progressively as the scores increased from 0 to 2, 3 to 5, and 6 to 8 points with the following percentages: 0% (*n* = 0/26), 2.9% (*n* = 2/68), and 69.2% (*n* = 9/13), respectively (Goodman & Kruskall Gamma *p* < 0.001, Fig. 4).

**Table 3:** Predictive model for adenoma gallbladder polyp: points assigned to each variable (0 ~ 8 points)

Variable	Comments	Points
Age > 55 years	Presence	3
	Absence	0
Bile viscosity < 7.5 s-^1^	Presence	3
	Absence	0
Bile cholesterol < 414.5 mg/dl	Presence	2
	Absence	0

## Discussion

In this study, we proposed a predictive model of adenomatous gallbladder polyps and found that low viscosity less than 7.5 s^− 1^ with an OR of 22.539, low bile cholesterol less than 414.5 mg/dl with an OR of 10.004, and age older than 55 years with an OR of 23.550 were predisposing factors for adenomatous polyps. The predictive scoring model using these factors showed high adherence with a sensitivity of 90.9% and specificity of 80.2% and an area under the curve of 0.845. Our results seemed superior to the previously reported quantitative contrast-enhanced ultrasound sensitivity and specificity parameters of 76.5 and 75%, respectively, reported by *Bae* et al. [10], or 68.1 and 70.2%, respectively, reported for differentiation based on polyp size over 1 cm in a study by *Wennmacker* et al. [11].

Bile acid is synthesized in the liver and its conjugation and transportation occur through the enterohepatic circulation. After that, the transformed bile acid is secreted into the bile and stored in the gallbladder. If there is abnormal bile secretion or functional impairment of the gallbladder, the bile contents stored in the gallbladder will change accordingly [12]. Several studies [13, 14] have reported that dyslipidemia, such as high low-density lipoprotein, would decrease gallbladder sensitivity to cholecystokinin and inhibit cholecystokinin secretion, resulting in a reduction of gallbladder motility and alteration of the mucosal esterification of free sterols from bile. This physiological change in the hepatic cholesterol mechanism could then promote cholesterol polyp formation. *Zhao* et al. [6] reported that the accumulation of cholesterol esters in epithelial cells of the submucosa of the gallbladder is thought to be a key factor in this association. The accumulated substances in the gallbladder submucosa are phagocytized by macrophages and generate foam cells. Then, finally, the surface of the gallbladder mucosa is converted into a polyp-like appearance with swollen villi. Our results showed that the level of bile cholesterol was a very useful marker for the differentiation of polyps.

Interestingly, in this study, a significant low viscosity was seen in the adenomas. Bile viscosity can be useful for examining the characteristics of bile, but there have been few reports on human bile viscosity. During storage in the gallbladder, mucus, a highly viscous substance, is secreted from goblet cells in the epithelium of the gallbladder. *Mucus contains mucins, a family of large glycosylated proteins, and bile viscosity is drastically increased by mucus secretion.* Previously, *Yoo* et al. [4] reported a relationship between cholesterol-related gallbladder disease and expression of the gallbladder mucin (MUC) gene in gallbladder epithelial cells. The expression of MUC3 and MUC5B genes was higher in cholesterol polyps than normal tissue of gallbladder and the upregulated expression of MUC genes contributed to the hypersecretion of mucin and the increased mucin in bile consecutively, led to increases in bile viscosity. Although we did not measure mucin levels, we believe that the high elevation in bile viscosity due to this characteristic mucin secretion process in cholesterol polyps can be useful in distinguishing it effectively from adenomatous polyps.

Additionally, increased age was also a significant predisposing factor for adenomatous polyps in the current study. Aging is closely related to changes in lipid profiles. *Cha* et al. [15] reported that ages of 65 years or older were correlated with neoplastic or malignant GBPs and our results showed that 56 years or older was a potent parameter in the predictive scoring model. The cutoff value of 55-year-old is noteworthy, which is relatively younger than the cutoff value commonly mentioned in previous studies. Given that our study consisted of a small sample size, the results need to be confirmed by a study with a larger number of samples.

This novel scoring system for distinguishing adenomas and cholesterol polyps could have clinical significance. Adenomas have malignant potential that generally follows the adenoma-carcinoma sequence, similar to that of colorectal cancer [16]. Gallbladder malignancy has been regarded as aggressive cancer with a dismal prognosis of 5–12% 5-year survival, but its survival could be prolonged to 80% if it could be detected with in situ disease [17]. These characteristic differences in the survival rate of gallbladder cancer patients according to the time of diagnosis emphasizes the importance of early detection of adenomatous polyps to prevent the spread the tumor cells or invade adjacent organs. In contrast, cholesterol polyps, which comprise most of the GBPs, have benign characteristics until they become symptomatic. Thus, the accurate discrimination of cholesterol polyps from adenomas can help optimize the indications for cholecystectomy for GBP and avoid unnecessary surgery, thus reducing the burden of medical costs and avert risks related to surgery, such as bile duct injury or bleeding.

To date, the most widely accepted indication for surgery for GBP is the diameter of polypoid lesions exceeding 10 mm based on ultrasonographic findings [5, 18]. Although we agree that these criteria are comparatively valid, it seems unreasonable to simply determine the malignant potential of GBP using this criterion only and use it to decide whether to perform surgery. Sonograms may vary in accuracy depending on the sonographer’s proficiency and there might be a discrepancy between the actual polyp size and the size measured by sonographic imaging. In addition, there are other reports that the 1 cm surgical threshold for GBP has insufficient diagnostic accuracy [11]. To overcome these limitations, several diagnostic modalities have been reported, but computed tomography or magnetic resonance imaging have also been reported to be unreliable in differentiating benign from premalignant lesions [19]. Therefore, our proposed discrimination method that reflects age and the biochemical characteristics of bile can reflect the nature of polyps more than size-based discrimination methods that merely reflect morphological aspects. It will help facilitate precise therapeutic decision-making and establish precise guidelines for the management of GBP.

Despite interesting outcomes, this study had some limitations that require attention to interpret. As described in the methodology section, only patients with GBPs with a size of 1 cm or more who underwent cholecystectomies were enrolled and those with GBPs less than 1 cm who underwent regular follow-up surveillance without surgery were not included. Therefore, this study did not represent all GBP patients and limits the application of the prediction model to the whole population of patients with GBPs. However, there is no general disagreement regarding surgery for GBPs of 1 cm or more and it is still the most common indication for surgical intervention. Our results, therefore, can provide practical guidance that is useful for those who are considering clinical intervention. Finally, our data had selection bias and some clinicopathologic data were incomplete because it was a retrospectively designed study. However, the selection bias could be alleviated because we prospectively performed sampling and collected data for all patients operated on due to GBP. A prospectively designed study involving a large number of adenomatous and cholesterol polyps is required in the next study. Also, we need to register and analyze all cases of GBP having sonographic evidence, regardless of the size of the GBP, to establish guidelines for the entire GBP population.

## Conclusions

Our results showed that high levels of bile cholesterol and viscosity associated with age ≤ 55 were significantly higher in patients with cholesterol polyps than in patients with adenomatous polyps and the novel scoring system using these values demonstrated high accuracy and specificity. It will be helpful for clinicians to set precise treatment guidelines for patients with GBPs that enable them, not only to detect adenomatous polyps earlier but also to avoid unnecessary surgery for cholesterol polyps.

## Data Availability

The research data that support the findings of this study are available on reasonable request from the corresponding author. The research data are not publicly available due to privacy or ethical restrictions.

## Abbreviations

GBP: Gallbladder polypoid lesion
TG: Triglycerides
ROC: Receiver-operating characteristic
OR: Odds ratio
MUC: Gallbladder mucin

## References

[1] Wu T, Sun Z, Jiang Y, Yu J, Chang C, Dong X (2019). Strategy for discriminating cholesterol and premalignancy in polypoid lesions of the gallbladder: a single-Centre, retrospective cohort study. ANZ J Surg.

[2] Gurusamy KS, Abu-Amara M, Farouk M, Davidson BR (2009). Cholecystectomy for gallbladder polyp. Cochrane Database Syst Rev.

[3] Goetze TO (2015). Gallbladder carcinoma: prognostic factors and therapeutic options. World J Gastroenterol.

[4] Yoo KS, Choi HS, Jun DW, Lee HL, Lee OY, Yoon BC (2016). MUC expression in gallbladder epithelial tissues in cholesterol-associated gallbladder disease. Gut Liver.

[5] Wiles R, Thoeni RF, Barbu ST, Vashist YK, Rafaelsen SR, Dewhurst C (2017). Management and follow-up of gallbladder polyps : joint guidelines between the European Society of Gastrointestinal and Abdominal Radiology (ESGAR), European Association for Endoscopic Surgery and other interventional techniques (EAES), International Society of Digestive Surgery - European federation (EFISDS) and European Society of Gastrointestinal Endoscopy (ESGE). Eur Radiol.

[6] Zhao MF, Huang P, Ge CL, Sun T, Ma ZG, Ye FF (2016). Conjugated bile acids in gallbladder bile and serum as potential biomarkers for cholesterol polyps and adenomatous polyps. Int J Biol Markers.

[7] Abel LL, Levy BB, Brodie BB, Kendall FE (1952). A simplified method for the estimation of total cholesterol in serum and demonstration of its specificity. J Biol Chem.

[8] Talalay P (1960). Enzymic analysis of steroid hormones. Methods Biochem Anal.

[9] Jüngst D, Lang T, von Ritter C, Paumgartner G (1991). Role of high total protein in gallbladder bile in the formation of cholesterol gallstones. Gastroenterology..

[10] Bae JS, Kim SH, Kang HJ, Kim H, Ryu JK, Jang JY (2019). Quantitative contrast-enhanced US helps differentiating neoplastic vs non-neoplastic gallbladder polyps. Eur Radiol.

[11] Wennmacker SZ, van Dijk AH, Raessens JHJ, van Laarhoven CJHM, Drenth JPH, de Reuver PR (2019). Polyp size of 1cm is insufficient to discriminate neoplastic and non-neoplastic gallbladder polyps. Surg Endosc.

[12] Hegade VS, Speight RA, Etherington RE, Jones DE (2016). Novel bile acid therapeutics for the treatment of chronic liver diseases. Ther Adv Gastroenterol.

[13] Kakimoto T, Kanemoto H, Fukushima K, Ohno K, Tsujimoto H (2017). Effect of a high-fat-high-cholesterol diet on gallbladder bile acid composition and gallbladder motility in dogs. Am J Vet Res.

[14] Zheng W, Zhang YJ, Bu XT, Guo XZ, Hu DY, Li ZQ (2017). LDL-cholesterol goal attainment under persistent lipid-lowering therapy in Northeast China: subgroup analysis of the dyslipidemia international study of China (DYSIS-China). Medicine (Baltimore).

[15] Cha BH, Hwang JH, Lee SH, Kim JE, Cho JY, Kim H (2011). Pre-operative factors that can predict neoplastic polypoid lesions of the gallbladder. World J Gastroenterol.

[16] Castells A (2015). Choosing the optimal method in programmatic colorectal cancer screening: current evidence and controversies. Ther Adv Gastroenterol.

[17] Amin MB, Greene FL, Edge SB, Compton CC, Gershenwald JE, Brookland RK (2017). The eighth edition AJCC Cancer staging manual: continuing to build a bridge from a population-based to a more "personalized" approach to cancer staging. CA Cancer J Clin.

[18] Mainprize KS, Gould SW, Gilbert JM (2000). Surgical management of polypoid lesions of the gallbladder. Br J Surg.

[19] Jang JY, Kim SW, Lee SE, Hwang DW, Kim EJ, Lee JY (2009). Differential diagnostic and staging accuracies of high resolution ultrasonography, endoscopic ultrasonography, and multidetector computed tomography for gallbladder polypoid lesions and gallbladder cancer. Ann Surg.

